# Psychopathology—a Precision Tool in Need of Re-sharpening

**DOI:** 10.3389/fpsyt.2018.00446

**Published:** 2018-09-19

**Authors:** Frauke Schultze-Lutter, Stefanie J. Schmidt, Anastasia Theodoridou

**Affiliations:** ^1^Department of Psychiatry and Psychotherapy, Medical Faculty, Heinrich-Heine University, Düsseldorf, Germany; ^2^Department of Clinical Psychology and Psychotherapy, University of Bern, Bern, Switzerland; ^3^Department of Psychiatry, Psychotherapy and Psychosomatics, Psychiatric Hospital, University of Zürich, Zürich, Switzerland

**Keywords:** descriptive psychopathology, clinical psychopathology, theoretical psychopathology, neuroscience, self-experiences, mind, brain, machine-learning

## Abstract

Psychopathology is the scientific exploration of abnormal mental states that, for more than a century, has provided a *Gestalt* for psychiatric disorders and guided clinical as well as scientific progress in modern psychiatry. In the wake of the immense technical advances, however, psychopathology has been increasingly marginalized by neurobiological, genetic, and neuropsychological research. This ongoing erosion of psychiatric phenomenology is further fostered by clinical casualness as well as pressured health care and research systems. The skill to precisely and carefully assess psychopathology in a qualified manner used to be a core attribute of mental health professionals, but today's curricula pay increasingly less attention to its training, thus blurring the border between pathology and variants of the “normal” further. Despite all prophecies that psychopathology was doomed, and with neurobiological parameters having yet to show their differential-diagnostic superiority and value for differential indication, psychiatric diagnosis continues to rely exclusively on psychopathology in DSM-5 and ICD-11. Their categorical systematic, however, is equally challenged, and, supported by advances in machine learning, a personalized symptom-based approach to precision psychiatry is increasingly advocated. The current paper reviews the objectives of psychopathology and the recent debate on the role of psychopathology in future precision psychiatry—from guiding neurobiological research by relating neurobiological changes to patients' experiences to giving a framework to the psychiatric encounter. It concludes that contemporary research and clinic in psychiatry do not need less but rather more differentiated psychopathologic approaches in order to develop approaches that integrate professional knowledge and patients' experience.

The term “psychopathology,” from the Greek ψυχ$\overset{´}{\eta }$ (psyche) for “soul” or “spirit,” π$\overset{´}{\alpha }$θoς (pathos) for “suffering,” and λoγóτυπα (logos) for “reason,” “discourse” or “opinion,” roughly translates into “teachings of the sufferings of the soul” and was coined in 1878 by the German psychiatrist Hermann Emminghaus (1). Yet, as a scientific discipline, psychopathology is commonly agreed to have started only 35 years later, in 1913 with the publication of Karl Japers' book “Allgemeine Psychopathologie” [(2), English: “General Psychopathology”] (3–10). To Jaspers, the subject of psychopathology was broadly “the individual as a whole in his illness, as far as it is a mental and psychogenic illness” and “the soul of the individual,” respectively [(8), p. 845]. With it, psychopathology had become the core science in psychiatry that, over the past century, has provided a “Gestalt” for psychiatric disorders and successfully guided clinical as well as scientific progress in psychiatry and clinical psychology (5, 6, 11). Yet, in the wake of tremendous technological advances, within recent decades, psychopathology has been increasingly eclipsed by neurobiological approaches in both research and teaching (6, 8, 11, 12). In the following, we will therefore revisit the objectives of psychopathology and discuss their current state and their potential future role.

## Objectives of psychopathology

In line with Jaspers' general definition, psychopathology is currently broadly defined, e.g., as “the scientific exploration of abnormal mental states” [(6), p. S147], “the subject matter of psychiatry, that is, pathologies of the psyche” [(9), p. 559], or “the discipline that assesses and makes sense of abnormal human subjectivity” [(10), p. 169]. Yet, despite this seeming agreement, the specific objectives of psychopathology and—consequently—its role in current and future psychiatric work and research still lack common understanding in psychiatry (6, 8). In 2010, the Task Force on Nosology and Psychopathology of the World Federation of Societies of Biological Psychiatry (WFSBP) thus reviewed approaches, theories, tasks, and tools of psychopathology in order to identify the main objectives of psychopathology in order to evaluate its role in the twenty-first century (8). It identified three main objectives of interrelated and partly interdependent tasks—descriptive, clinical and theoretical psychopathology (8). Illustrated by examples from (early) psychosis research, these are outlined and discussed in the following.

## Descriptive psychopathology

The main tasks of descriptive or general psychopathology are two-fold (8). The first task is to describe and to denominate persons' subjective experiences and behaviors in a way that allows objective communication about them that is free of personal, cultural and school-specific interpretations (8–10). In doing so, ideally, psychopathology would provide a shared language (10). Yet, some of the critique on general psychopathology might in fact stem from the failure of the psychopathological community to provide, maintain, and impart an unambiguous nomenclature, i.e., from the lack of “a common language in the “Tower of Babel” that science has become” [(8), p. 848].

The early detection and intervention in psychoses is one such recent field of research in psychiatry in that such a persistent Babylonian speech confusion and its effects can be observed (13, 14). For example, in a recent critique of the ultra-high risk (UHR) approach (15), the majority of arguments were based on the assumed equality of UHR-relevant attenuated psychotic symptoms (APS) assessed by clinicians in patient samples using specific semi-standardized instruments and “psychotic-like experiences” assessed in the community by lay-persons using fully standardized instruments or by self-rating instruments (13, 14). In the critique (15), positive results in both assessment modes were equally regarded as “slightly-but-not quite psychotic,” “low-grade psychotic symptoms,” or “psychotic experiences,” albeit studies showing that such community measures commonly overestimate the presence of clinician-assessed APS to a degree that casts serious doubts on their comparability (14, 16–18). This lack of psychopathological understanding and the resulting falsely assumed equality of phenomena of similar names (13, 14) then paved the way to unfounded—if not, from a clinical point of view, absurd—conclusions (14) such as “psychotic experiences” merely being “a marker for the severity of non-psychotic states” [(15), p.201].

However, Babylonian speech confusion is not restricted to wrongly assuming phenomenological equality by using similar terms interchangeably (13) but also includes using different terms for equal phenomena—often in the context of different schools. An example of this in psychosis literature is the current nomenclature of patients' self-descriptions of deviations in their mental processes. While these, within a biomedical theoretical framework, are known as “basic symptoms” (19–23), within the framework of the phenomenological tradition in philosophy, they are called “self-disturbances,” “anomalous self- or subjective experiences” and “self-disorders” or “anomalous world experiences” (21, 22, 24–26). Thus, already in light of such imponderability of nomenclature, it might not be all surprising that, in psychiatry and related fields, researchers and clinicians alike increasingly seek rescue in neuroscience and its more definite terms and palpable constructs.

The second task of general psychopathology, including “special” and “functional” psychopathology, is to distinguish “abnormal” from “normal” experiences and behaviors and to describe the nature and developments of these “symptoms” (8). In special psychopathology, the focus has been on patients' self-experiences and self-reports of their mental states rather than on their observable expressions and behaviors that are regarded as important but less specific than personal experiences in terms of first-person perspective narratives (5, 6, 9). Functional psychopathology adopted a different strategy by defining deviant mental functions by clinicians' or third-persons' observations either of persons' expressions and behaviors or by their test performances, e.g., in neuropsychological tasks (8). With regard to test performances, functional psychopathology commonly defines the “abnormal” by standard deviations from the mean or similar measures, e.g., in case of the construct “intelligence” or of the 6th dimension, “cognitive impairment” of the severity assessment of symptoms of psychosis introduced in DSM-5 (27).

Traditionally, focus in psychiatry has been on special psychopathology. Yet, in the development of criteria-based operational diagnoses in DSM and ICD, emphasis was laid on interrater reliability rather than validity. Therefore, functional psychopathology was given a more prominent role along with the use of fully structured and sometimes even fully standardized instruments in special psychopathology (5, 6, 8, 12). In special psychopathology, however, empathy has been the main clinical tool to recreate and understand the patients' self-experience and to shape the psychiatric encounter. Thereby, the clinician systematically explores patients' self-experiences and translates these and certain accompanying aspects of their expression and behavior into specific predefined symptoms (5, 6, 9). Such an empathetic, understanding approach does not rule out the biomedical approach of seeing abnormal phenomena as symptoms whose underlying dysfunctions are to be cured but rather compliments it by exploring patients' meaning of their symptoms (9, 10). Besides, it does not rule out the use of semi-structured interviews to ensure addressing all relevant symptoms, including interviews specifically addressing self-experienced “abnormalities” (25, 26, 28, 29). Yet, this should be done in a conversational, context-sensitive way that supports giving a *Gestalt* to the patient's complaints (5, 9, 10, 12). The *Gestalt* arising from a careful, detailed and thorough exploration is more than simply an aggregation of symptoms—just as the picture arising from a jigsaw puzzle is more than the sum of its pieces; it is a coherent picture of the patient's mind and the foundation of a valid clinical and diagnostic appraisal.

The skill to precisely and carefully assess special psychopathology in a qualified manner used to be a core attribute of mental health professionals, but today's curricula pay increasingly less attention to its time-consuming training (5, 9, 12, 30). As a consequence, the border between pathology and variants of the “normal,” whose supposed lack of clear definition is often held against special psychopathology, is further blurred. This ongoing erosion of the core skill of psychiatry is further fostered by clinical casualness as well as (economically) pressured health care and research systems (9) and endeavors such as the Research Domain Criteria (RDoC) project, whose aim is to classify “mental disorders based on dimensions of observable behavior and neurobiological measures” using big data approaches [(31), p.1205].

## Clinical psychopathology

Based on the descriptive psychopathology, nosological, or clinical psychopathology identifies symptoms that are significant to supposed nosographical distinctions and groups them together in nosographical systems, i.e., syndromes and diagnostic entities (8–10)—nowadays, increasingly with help of some kind of statistical modeling (8). In doing so, it has laid the grounds for studies into the causes of mental disorders, i.e., etiological psychopathology, and to the development of evidence-based treatment guidelines (8). Yet, mental disorders neither exist per se nor are they distinct diagnostic entities, but commonly share features. In mental health research, however, shared features are not only problem of clinical psychopathology but also of genetics (32) and neurobiology (33, 34).

Furthermore, these diagnostic entities in terms of mental disorders are not merely defined by a selected group of symptoms. In addition, they generally make objective requirements on symptoms' course, such as their minimum time of persistence (thus including some consideration of the crucial dimension of time (6)), their presentation (such as current frequency of occurrence or association–or lack of it–with certain situations or life events) and their non-functionality. Thus, a certain self-experience might only become a “symptom” in a pathological sense of meaning under certain circumstances, and symptoms and syndromes or disorders should not be equaled in discussions, such as in the exemplary critique of the UHR approach (14, 15). The presence of a clinician-assessed APS does not constitute presence of a UHR criterion that, in addition, makes requirements on its frequency and course as well as consideration of the context in that it occurs (14, 35–37). For this reason, roughly only one of ten persons of the community who had reported APS in a clinician-conducted interview actually met UHR criteria (35). Thus, some of the problems of distinguishing “normal” mental states from “abnormal” mental disorder might not pertain to the definition of phenomena but rather to their frequency and persistence allowances in diagnostic criteria, which are often not decided based on data but expert consensus.

Furthermore, current diagnostic manuals–and recent approaches toward a dimensional nosology such as the Hierarchical Taxonomy Of Psychopathology (HiTOP) initiative (38, 39)–only rely on a fraction of possible symptoms that merely convey a fragmented picture of a patient's mind and whose selection was mainly driven by reliability rather than validity aspects (5, 12). Today's psychiatric training, however, is focusing on only diagnosis-relevant symptoms (9, 10, 12, 30) that are frequently observable epiphenomena arising from patient's coping (incl. the search for meaning and explanation) with different, often distressing self-experiences (5, 30). These symptoms, however, had been initially intended only as gatekeepers in terms of the minimum symptoms needed to make a diagnosis rather than perceived as a conclusive description of symptoms associated with the respective diagnosis (12). Thus, in the wake of the introduction of DSM and ICD, “classics of psychopathology are now largely ignored” and “research in psychopathology is a dying (or dead) enterprise” [(12), p. 111]; and clinicians are discouraged from getting to know the individual patient and from understanding the *Gestalt* of his or her state of mind (5, 9, 10, 12, 30). Yet, as one commonly only finds what one is looking for, findings of a study examining case notes for descriptions of basic symptoms (40) are not surprising. Basic symptoms, which represent a prototype of patients' self-experiences but are completely unconsidered in diagnostic criteria of DSM and ICD, were 16 times more likely reported by patients with psychotic disorder in a special interview (28) than they were recorded in their case notes (40).

## Theoretical psychopathology

Exceeding, yet depending on and interacting with both descriptive and clinical psychopathology, theoretical psychopathology is the study of etiology or pathogenesis and, thus, strongly links psychopathology to neuroscience ((8); see also ‘psychopathology in neuroscience and precision psychiatry’ below). It lends some validity to mental syndromes but also provides the foundations for etiological and pathogenetic research (8). However, with sacrificing validity to reliability in DSM, the research targets provided by its diagnoses might be the wrong ones (12), thus gradually weakening the cause of psychopathology. Furthermore, while theoretical psychopathology borrowed methods from other science that were subordinated to the needs of psychopathological research in the past, with immense advances in neuroscience and genetics, these increasingly took a life on their own, thereby losing their already weakened psychopathological foundation (8). Thus, with increasing separation of neuroscience and genetics from the very subject of psychiatry—i.e., the mind, the “dehumanizing impact” of DSM on the practice of psychiatry [(12), p. 111] will be multiplied.

## Psychopathology in neuroscience and precision psychiatry

Psychiatry and psychopathological research, in particular theoretical psychopathology, has always been positioned on a continuum between (natural) facts and (human) constructs (8, 10). This is a unique, yet often forgotten feature of psychiatry within biomedical science; and it is the mind rather than “brain events by themselves” that is of interest in psychiatry (5, 8, 10). Yet, this intermediate position has always fueled discussions on the role and value of psychopathology between advocates of different positions on this continuum, e.g., between neuroscientists located on the pole of natural facts and philosophers located on the pole of human constructs, who often lack a common language (8). Thus, with the two positions drifting further and further apart in the process of increasing specialization in science, psychopathology will have to make their re-integration, i.e., the reintegration of “mind” and “brain,” its explicit objective (8, 10).

In order to reach these aims, the still widely accepted limited range of symptoms currently considered in diagnostic criteria and the structures of diagnosis need to be broadened again to the full range of patients' self-experiences in descriptive psychopathology and reassessed in clinical psychopathology (5, 10–12). The same applies to specific concepts of properties of the “psyche” or symptoms that often have been established decades ago without subsequent examination of their nowadays' social, historical and philosophical appropriateness and that are often used without sufficient knowledge of their origin and historical development (11, 13). Thus, today, neuroscientists and geneticists who try to find the neurobiological or genetic correlates of mental disorders or properties are faced with likely inadequate concepts of mental disorders. Additionally, they often lack understanding of the conceptual basis of their target (disorder or property) and the validity of its assessment methods; and rarely have a team member, e.g., a psychopathologist, able to expertly address these matters (11).

In the last few years, machine learning has made huge progress as a statistical method to classify and predict human experiences and behavior, incl. mental disorders, based on interpreting different kinds of information (41–44). Compared to more traditional prediction methods (such as regression analyses), predictive accuracy with machine learning methods (such as Support Vector Machines, Random Forests, and Deep Learning) increases from 30 to 40% to frequently over 90% in individualized prediction that is the basis of precision psychiatry (45). The use of these methods, however, is not restricted to neuroscience—in that it is currently mainly used—but can well be applied in psychopathology. In fact, it was recently shown that clinical variables can perform as well as neurobiological ones (46). Furthermore, other next generation statistical approaches such as joint modeling, time series analyses or network models might help identifying valid syndromes of little overlap if not based only on the “small world” of symptoms currently included in the definition of mental disorders (47, 48). Promising to this aim are patients' self-experiences in terms of basic symptoms and anomalous self- or world experiences, respectively (5, 20–23, 28, 29). These, in particular basic symptoms, have the unbeatable advantage to clearly distinguish “normal” from “abnormal” experiences as, by definition, they differ from what patients consider to be their normal mental self and functions. These subtle disturbances in any kind of mental process (e.g., stress tolerance, drive, affect, thinking, speech, perception, motor action, and central-vegetative functions) are self-experienced with immediate and full insight into their abnormal nature, and reported by patients as “abnormalities” (20–23, 28, 29).

For all these different aspects to be considered, the different specialties involved in this endeavor (incl. psychiatry, psychology, neuroscience, philosophy, genetics, epidemiology, computer science, and mathematics) will have to closely work together on equal footing in order to succeed in understanding the underpinnings and mechanisms of mental disorders. Developing a clear common language and bringing together their particular skills and expertise, multidisciplinary projects are needed:

- to validly define on a basic, fine-grained level what is “abnormal” and “normal” both in human experience and in brain function (10),
- to develop valid, reliable, broadly applicable, and sufficiently economic instruments for the assessment of such “abnormalities” whose assessment time should be fully covered by health insurances—this bringing in health politics (11),
- to detect if and how these “abnormalities” and “normalities” relate to each other over time (6, 10, 11),
- to newly define valid and reliable nosological constructs, i.e., syndromes or mental disorders, based on these “abnormalities” (5, 6, 8, 10, 12, 49),
  ◦ that are also in terms with and perceptive about the full range of complaints with that patients will present themselves (5, 6, 10, 12, 49),
  ◦ allow precise personalized diagnosis and prognosis (6),
  ◦ for that efficient benign treatments exist or can be developed, likely as modular therapies (49),
- to include the dimension of time, i.e., the likely course of symptoms, in order to not only classify and treat the current mental state (“Zustandsbild”) but the underlying illness (“Krankheit”) and its psychosocial functional consequences (6).

## Conclusion

Psychopathology is currently a neglected, if not dying science—not least, because current concepts of mental disorders failed to produce adequate neurobiological and genetic targets. Yet, as we demonstrated, problems within the field of psychopathology and the resulting neglect of psychopathology have brought about this failure. Thus, contemporary research and clinic in psychiatry do not need less but rather more differentiated psychopathologic approaches in order to develop approaches that integrate professional knowledge and patients' self-experience and offer more appropriate valid targets for neurobiological and genetic research than the broad, rather ill-defined constructs that definitions of mental disorders currently represent, i.e., as Nancy Andreasen has pointed out repeatedly over more than 20 years [(11), p. 112]:

“We need to make a serious investment in training a new generation of real experts in the science and art of psychopathology. Otherwise, we high-tech scientists may wake up in 10 years [that is, now; comment by authors] and discover that we face a silent spring. Applying technology without the companionship of wise clinicians with specific expertise in psychopathology will be a lonely, sterile, and perhaps fruitless enterprise.”

## Author contributions

FS-L was responsible for the conception of the work and drafted the first version of this work. SS and AT revised it critically for important intellectual contents. All authors provided approval for publication of the content.

### Conflict of interest statement

The authors declare that the research was conducted in the absence of any commercial or financial relationships that could be construed as a potential conflict of interest.
